# Lung microbiota associations with clinical features of COPD in the SPIROMICS cohort

**DOI:** 10.1038/s41522-021-00185-9

**Published:** 2021-02-05

**Authors:** Kristopher Opron, Lesa A. Begley, John R. Erb-Downward, Christine Freeman, Siddharth Madapoosi, Neil E. Alexis, Igor Barjaktarevic, R. Graham Barr, Eugene R. Bleecker, Russell P. Bowler, Stephanie A. Christenson, Alejandro P. Comellas, Christopher B. Cooper, David J. Couper, Claire M. Doerschuk, Mark T. Dransfield, MeiLan K. Han, Nadia N. Hansel, Annette T. Hastie, Eric A. Hoffman, Robert J. Kaner, Jerry Krishnan, Wanda K. O’Neal, Victor E. Ortega, Robert Paine, Stephen P. Peters, J. Michael Wells, Prescott G. Woodruff, Fernando J. Martinez, Jeffrey L. Curtis, Gary B. Huffnagle, Yvonne J. Huang

**Affiliations:** 1 Division of Pulmonary/Critical Care Medicine, University of Michigan, Ann Arbor, MI USA; 2 Research Service, VA Ann Arbor Healthcare System, Ann Arbor, MI USA; 3 University of North Carolina at Chapel Hill, Chapel Hill, NC USA; 4 University of California at Los Angeles, Los Angeles, CA USA; 5 Columbia University, New York, NY USA; 6 University of Arizona College of Medicine, Tucson, AZ USA; 7 National Jewish Health, Denver, CO USA; 8 University of California at San Francisco, San Francisco, CA USA; 9 University of Iowa, Iowa City, IA USA; 10 University of Alabama at Birmingham, Birmingham, AL USA; 11 John Hopkins University, Baltimore, MD USA; 12 Wake Forest School of Medicine, Winston-Salem, NC USA; 13 Weill Cornell Medical College, New York, NY USA; 14 University of Illinois, Chicago, IL USA; 15 University of Utah, Salt Lake City, UT USA; 16 Medical Service, VA Ann Arbor Healthcare System, Ann Arbor, MI USA; 17 Department of Molecular, Cellular and Developmental Biology, University of Michigan, Ann Arbor, MI USA

**Keywords:** Microbiome, Health care

## Abstract

Chronic obstructive pulmonary disease (COPD) is heterogeneous in development, progression, and phenotypes. Little is known about the lung microbiome, sampled by bronchoscopy, in milder COPD and its relationships to clinical features that reflect disease heterogeneity (lung function, symptom burden, and functional impairment). Using bronchoalveolar lavage fluid collected from 181 never-smokers and ever-smokers with or without COPD (GOLD 0-2) enrolled in the SubPopulations and InteRmediate Outcome Measures In COPD Study (SPIROMICS), we find that lung bacterial composition associates with several clinical features, in particular bronchodilator responsiveness, peak expiratory flow rate, and forced expiratory flow rate between 25 and 75% of FVC (FEF_25–75_). Measures of symptom burden (COPD Assessment Test) and functional impairment (six-minute walk distance) also associate with disparate lung microbiota composition. Drivers of these relationships include members of the *Streptococcus, Prevotella, Veillonella, Staphylococcus*, and *Pseudomonas* genera. Thus, lung microbiota differences may contribute to airway dysfunction and airway disease in milder COPD.

## Introduction

Chronic obstructive pulmonary disease (COPD) is a predominantly smoking-related lung disease, characterized by airflow obstruction, local and systemic inflammatory responses, and phenotypic heterogeneity. Inflammation can persist after smoking cessation for reasons not fully understood, and patients vary in disease development, progression and other outcomes. Bacterial colonization of the airways in COPD has long been recognized from culture-based studies, but its role in the pathogenesis or progression of COPD remains unclear.

Recent studies, using culture-independent sequencing to profile microbial communities more comprehensively, have reported alterations in airway bacterial composition mostly in more severe COPD^1–5^. Less is known about the lung microbiome in milder COPD or in smokers without evidence of airflow obstruction and, in particular, whether differences in lung microbiota associate with clinical or biological features in earlier stages of COPD development. In the National Heart, Lung and Blood Institute (NHLBI) Subpopulations and Intermediate Outcome Measures in COPD study (SPIROMICS)^6^, 50% of current or former smokers without airflow obstruction reported significant respiratory symptoms, coupled with activity limitation and more exacerbation-like events^7^. These findings indicate a need to determine whether changes in the lung microbiome may contribute to clinical outcomes in milder COPD or in those who do not meet current spirometric criteria for the diagnosis.

Few studies of COPD have examined the lung microbiome sampled by bronchoscopy^8–13^, an invasive approach that may provide unique insights into the potential role of lung microbiota in COPD pathogenesis. Using bronchoalveolar lavage fluid (BAL) collected from subjects in the well-characterized SPIROMICS cohort^6,14^, we explored the hypotheses that differences in lung bacterial composition are associated with a diagnosis of COPD and/or with clinical features that reflect COPD pathophysiology, including measures of lung function and symptom burden.

## Results

### Cohort characteristics

Characteristics of the 181 subjects in this study are summarized in Table 1. Those with COPD were older and, as expected, had lower lung function and greater symptom burden [higher scores on COPD Assessment Test (CAT), and St. George’s Respiratory Questionnaire (SGRQ)]. Subjects with COPD also displayed greater lung function response to an inhaled bronchodilator, as measured by both change in FVC (forced vital capacity) or FEV_1_ (forced expiratory volume in one second); these and other lung function measurements, except for FEF_25–75_, did not differ significantly from measurements at the baseline SPIROMICS visit (Supplementary Table 1). Current smoking was associated with differences in CAT and SGRQ scores, but not lung function measures.

**Table 1:** Baseline characteristics of subjects.

	All (*n* = 181)	Never-smokers (*n* = 24)	Smokers, no COPD(FEV_1_/FVC > 0.70) (*n* = 79)	Mild/Moderate COPD(50%<FEV_1_ < 80%) (*n* = 71)	Severe COPD(FEV_1_ < 50%) (*n* = 7)	*p*-value^a^
Age (yrs)	61	55 (42, 71)	57 (41, 75)	65 (46, 77)	65 (42, 74)	<0.001
Sex (male %)	52%	33%	49%	63%	50%	0.06
Caucasian (%)	69%	67%	61%	70%	100%	0.05
Current smoker (%)	34%	N/A	43%	38%	17%	<0.001
Pack-yrs	–	0	35 (20, 113)	45 (23, 270)	43 (25, 90)	<0.001
FEV1/FVC	0.72	0.81 (0.72, 0.91)	0.77 (0.57, 0.89)	0.60 (0.34, 0.77)	0.52 (0.46, 0.67)	<0.001
FEV1 (post-BD, L)	2.6	2.9 (1.9, 2.2)	2.9 (1.4, 4.8)	2.2 (0.9, 3.9)	1.7 (1.3, 2.9)	<0.001
FEV1 (post-BD, %pred)	92.9	101.9 (72.5, 118.1)	98.9 (65.7, 133)	75.5 (41.1, 111.4)	54.9 (46.6, 86.6)	<0.001
FVC (post-BD, L)	3.7	3.4 (2.3, 5.4)	3.7 (2.4, 6.0)	3.8 (2.4, 6.9)	3.4 (2.7, 4.3)	0.73
FVC (post BD, %pred)	99.6	99.6 (71.6, 113.3)	99.9 (73.7, 129.6)	100.9 (67.1, 132.8)	80.6 (73.4, 94.9)	0.027
FEF25-75 (L/sec)	1.8	3.3 (1.6, 5.4)	2.6 (0.5, 5.2)	0.9 (0.3, 2.9)	0.6 (0.4, 1.5)	<0.001
Peak expiratory flow rate (L/sec)	7.2	8.8 (4.3, 11.3)	8.0 (2.8, 13.3)	6.3 (3.2, 12.4)	5.0 (4.1, 7.8)	<0.001
Bronchodilator response						
FEV_1_ (% change)	7.3	5.0 (−6.8, 13.6)	6.2 (−13.6, 33.5)	10.6 (−10.6, 34.2)	5.1 (2.1, 19.0)	<0.001
FVC (% change)	1.8	−0.6 (−11.9, 5.9)	0.5 (−17.7, 19.0)	6.4 (−8.0, 54.2)	9.9 (4.6, 31.2)	<0.001
Six-minute hall walk distance (meters)	462	490 (357, 645)	464 (114, 626)	450 (126, 740)	451 (360, 576)	0.35#
CAT score	8	2 (0, 16)	7 (0, 32)	10 (1, 35)	14 (2, 29)	<0.001#
SGRQ total score	16	4 (3, 35)	13 (3, 85)	25 (3, 83)	32 (18, 67)	<0.001#

Data are median or % (min, max). Data shown are from the annual visit prior to bronchoscopy except for 6-min walk (baseline visit only).

*FEV*_*1*_ forced expiratory volume in 1 sec, *FVC* forced vital capacity, *FEF*_*25–75*_ maximum mid-expiratory flow, *CAT* COPD Assessment Test, *SGRQ* St. George’s Respiratory Questionnaire.

^#^*p* < 0.05 by current smoking status.

^a^Kruskal–Wallis or Fisher’s exact test across all four groups.

### Lung bacterial composition is associated with specific measures of lung function, symptom burden and functional impairment

First, to examine whether variation between subjects in their overall lung bacterial composition (β-diversity) associated with COPD status and related clinical characteristics, we performed distance-based PERMANOVA using Bray-Curtis and weighted Unifrac distance measures, using data from all 181 subjects. This analysis identified significant associations between variation in lung microbiota composition and specific measures of lung function, symptom burden, and functional impairment (PERMANOVA *p* < 0.05; Table 2 and Supplementary Table 2). Although pre-bronchodilator measures of FEV_1_ and FVC were not associated with lung microbiota variation (not shown), measures of post-bronchodilator response (BDR), in particular change in FVC, were. FEF_25–75_ (forced expiratory flow rate between 25 and 75% of FVC) and PEFR (peak expiratory flow rate) also were associated with lung microbiota variation, as were CAT and SGRQ scores and baseline six-minute walk distance. This analysis did not identify associations between lung microbiota variation and a diagnosis of COPD, age, sex, current smoking, or inhaled corticosteroid use, nor with study center or season in which the bronchoscopy was performed. Collectively, these initial findings suggested that differences between subjects in their overall lung bacterial composition were more strongly linked to specific measures of airways dysfunction and related symptom burden, rather than a diagnosis of COPD per se.

**Table 2:** Clinical features associated with differences in lung bacterial community structure (N = 181 subjects).

Lung function and symptom-related outcomes		Bray–Curtis *p*	Weighted Unifrac *p*
FVC bronchodilator response (% change)		0.005	0.003
FVC bronchodilator response (mL change)		0.011	0.004
FEV1 bronchodilator response (% change)		0.013	0.032
FEV1 bronchodilator response (mL change)		0.044	0.085
Peak expiratory flow rate, pre-bronchodilator (L/sec)		0.033	0.079
Peak expiratory flow rate, post-bronchodilator (L/sec)		0.036	0.024
FEF25-75, pre-bronchodilator (L/sec)		0.051	0.043
FEF25-75, post-bronchodilator (L/sec)		0.061	0.048
COPD Assessment Test (CAT) score	0.038		0.020
SGRQ total score	0.014		0.017
SGRQ- impact domain	0.009		0.006
SGRQ- activity domain	0.052		0.039
6-min walk distance (meters)	0.003		0.003
6-min walk distance (% predicted)	0.011		0.006

*p*-values for distance-based PERMANOVA based on 1000 permutations. Variables demonstrating a significant association (*p* < 0.05) by either distance measure are shown. See also Supplemental Table E3. Lung function and symptom measures are from the annual visit preceding bronchoscopy; 6-min walk distance is from the baseline visit.

**Fig. 1:**
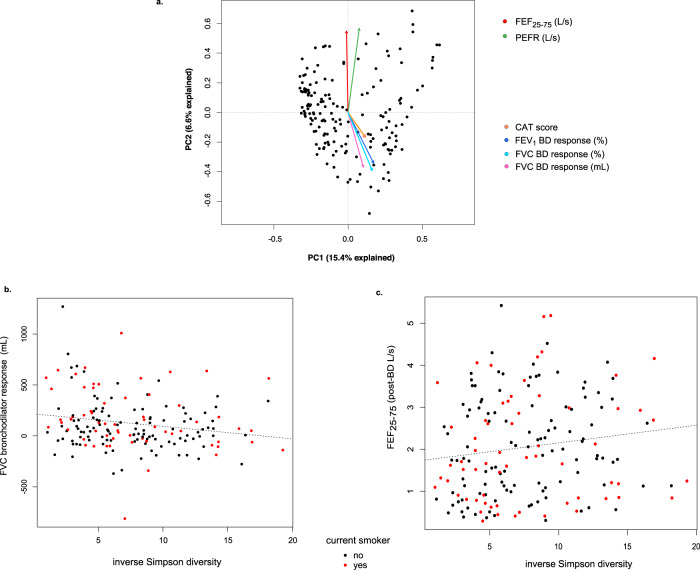
Variation in lung bacterial community composition is associated with specific measures of lung function and symptoms. **a** Principal component analysis (PCA) of the overall variation in BAL bacterial composition and relationships to the associated clinical measures for the entire cohort (*N* = 181). Contrasting relationships exist between lung microbiota and measures of airflow limitation (FEF_25–75_ and peak expiratory flow rate, PEFR) compared to that for measures of bronchodilator response and symptoms (COPD Assessment Test, CAT). PCA based on Hellinger transformation of OTU abundance counts. Vectors indicate direction and magnitude of the linear relationship between a clinical variable and the gradient of bacterial composition shown by PCA (R function *envfit*). **b**, **c** Correlations between within-sample bacterial diversity (inverse Simpson index) to FVC bronchodilator response and FEF_25–75_ (Spearman *r* = −0.26, *p* = 0.005 and *r* = 0.17, *p* = 0.02, respectively).

We next applied principal component analysis (PCA) to examine further the relationships between these associated clinical features and lung microbiota variation between subjects. This analysis demonstrated contrasting gradients of bacterial compositional difference related to BDR and CAT scores versus FEF_25–75_ and PEFR (Fig. 1A; R function *envfit*). CAT scores followed the same compositional gradient as BDR, and independently CAT score and FVC_BDR were correlated (Spearman R; *r*_s_ = 0.35, *p* < 0.001).

We also determined within-sample measures of bacterial diversity (inverse Simpson index). These correlated negatively with FVC_BDR (*r*_s_ = −0.26, *p* = 0.005) but positively with FEF_25–75_ (*r*_s_ = 0.17, *p* = 0.02; Fig. 1B, C), which support the converse relationships of these variables observed by PCA. This diversity measure did not differ by current smoking status, consistent with our above reported finding that current smoking was not associated with inter-subject variation in lung bacterial community structure.

### Analysis of ever-smokers with or without mild-moderate COPD

**Fig. 2:**
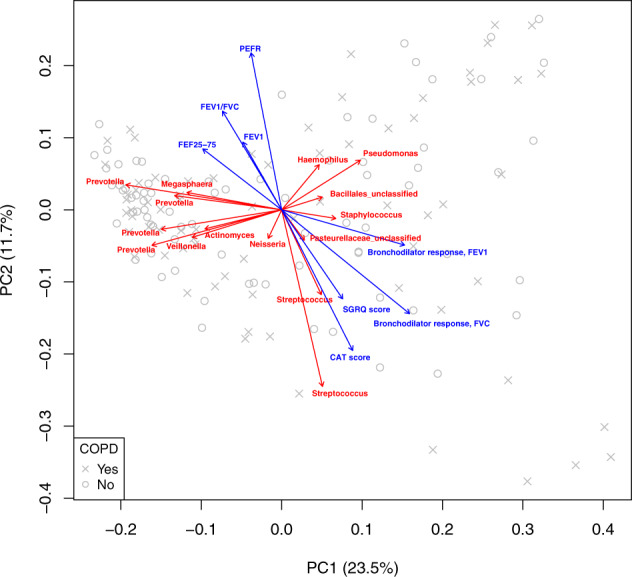
Differential relationships between lung bacterial composition, specific lung function measures, and symptoms are maintained among ever-smokers without or with mild-moderate COPD. Principal coordinate analysis (PCoA) of lung bacterial community variation (weighted Unifrac distance) among ever-smokers without (gray circles) or with (gray Xs) mild-moderate COPD and relationships to the clinical variables shown (blue arrows; R *envfit*). An overlaid biplot analysis shows the microbiota members (red arrows) driving the observed variation in lung bacterial community structure amongst these ever-smokers.

A crucial longstanding question is why only some smokers develop COPD. Exploiting the advantage that our cohort consisted primarily of ever-smokers (current or former) without COPD or with mild-moderate COPD, we next focused on these two groups to explore the potential role of differences in the lung microbiota in the disease process. We again observed associations between variation in lung bacterial composition and measures of BDR and 6-min walk distance (Unifrac distance-based PERMANOVA; *r*^2^ = 0.02 for both measures, *p* = 0.019 and *p* = 0.007, respectively), but not with FEV_1_/FVC ratio, FEV_1_ % predicted or COPD status itself. BDR measures were greater in the mild-moderate COPD group compared to ever-smokers without COPD (Wilcoxon rank-sum test, *p* < 0.001 for both change in FEV_1_ or FVC), while CAT scores were marginally higher in the COPD group (Wilcoxon rank sum *p* = 0.04). PCA again demonstrated contrasting gradients of lung bacterial composition associated with BDR versus other lung function measures, including PEFR and related parameters that reflect airflow obstruction (Fig. 2, blue arrows; Unifrac distance; R *envfit*). As before, similar directional gradients of bacterial composition were observed for FVC_BDR and CAT score, which remained correlated with each other (*r*_s_ = 0.30, *p* < 0.001). This directionality contrasted from the gradients associated with FEV_1_/FVC, FEV_1_, FEF_25–75_, and PEFR. Biplot analysis indicated that members of the *Streptococcus*, *Staphylococcus*, *Pseudomonas*, *Prevotella*, and others were key contributors to the variation in lung bacterial composition (Fig. 2, red arrows) among these ever-smokers without or with mild-moderate COPD.

**Fig. 3:**
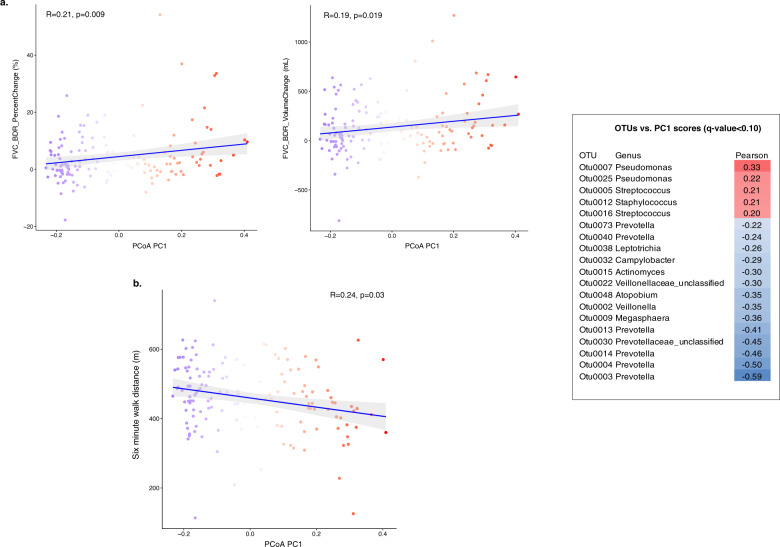
Post-bronchodilator FVC response and 6-min walk distance correlate with variation in lung bacterial composition along principal component 1, which explained 23.5% of the total variation in lung bacterial community structure among ever-smokers. Each dot represents an individual BAL sample and is colored according to its PC score. OTUs contributing significantly (*q*-value < 0.10) to PC1 are shown in the accompanying table, ranked by Pearson’s correlation coefficient. The relative abundances of *Prevotella, Pseudomonas*, *and Streptococcus* are primary drivers of PC1 variation. **a** Post-bronchodilator FVC response measured by change in percent and volume in mL. **b** Six-minute walk distance (meters).

We next examined each principal component axis to dissect further which particular bacteria and clinical measurements together contributed to the variation in bacterial community structure amongst subjects. For axis 1 (23.5% of the total variation) principal component (PC) scores were significantly correlated with two measures of FVC_BDR (% change in FVC; *R* = 0.21, *p* = 0.01; FVC volume response in milliliters, *R* = 0.19, *p* = 0.019; Fig. 3A), and with six-minute walk distance (*R* = –0.24, *p* = 0.003; Fig. 3B). Bacterial taxa contributing to PC1 scores included members of *Pseudomonas* (OTU0007 *R* = 0.33 *q* < 0.001; OTU0025 R = 0.22, *q* = 0.055), *Streptococcus* (OTU0005 *R* = 0.21, *q* = 0.06; OTU0016 *R* = 0.20, *q* = 0.097), *Staphylococcus* (OTU0012 *R* = 0.21, *q* < 0.001), and multiple *Prevotella* (OTU0003, OTU0004, OTU0014; *R* = −0.46 to −0.59; *q* < 0.001).

**Fig. 4:**
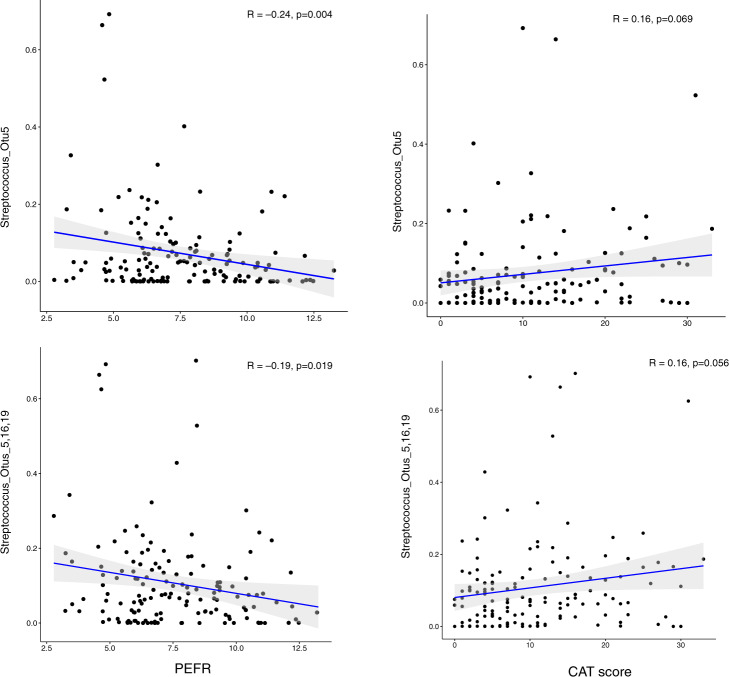
Streptococcus, PEFR and CAT score in ever-smokers with or without COPD. The relative abundance of *Streptococcus* Otu5, either alone (top panels) or together with other *Streptococcus* OTUs (bottom panels), correlate negatively with peak expiratory flow rate (PEFR) and positively with COPD Assessment Test (CAT) score.

Axis 2, which explained an additional 11.7% of lung bacterial community variation, was associated primarily with measures of PEFR (*R* = 0.20, *p* = 0.014). Five taxa were significantly correlated with PC2 scores (*q*-value <0.10). All were negative correlations, the strongest being with three *Streptococcus* members (OTU0005, *R* = −0.73, *q* < 0.001; OTU0016, *R* = −0.33, *q* = 0.002; OTU0019, *R* = −0.25, *q* = 0.049). *Streptococcus* relative abundance also displayed correlative relationships with PEFR, CAT score, as well as FEV_1_ and FEV_1_/FVC. OTU0005 had the strongest negative relationship with PC2, associated with lower PEFR, and trended weakly with higher CAT score (Fig. 4) and lower FEV_1_ (*R* = –0.14; p = 0.095). When the combined relative abundance of these Streptococci (OTUs 0005, 0016 and 0019) was considered, a weak negative trend with FEV_1_/FVC also was noted (*R* = −0.16, *p* = 0.081). Altogether these findings suggest that a collection of lung microbiota members contribute to the dysbiosis associated with airways dysfunction and symptoms observed in these ever-smokers.

**Fig. 5:**
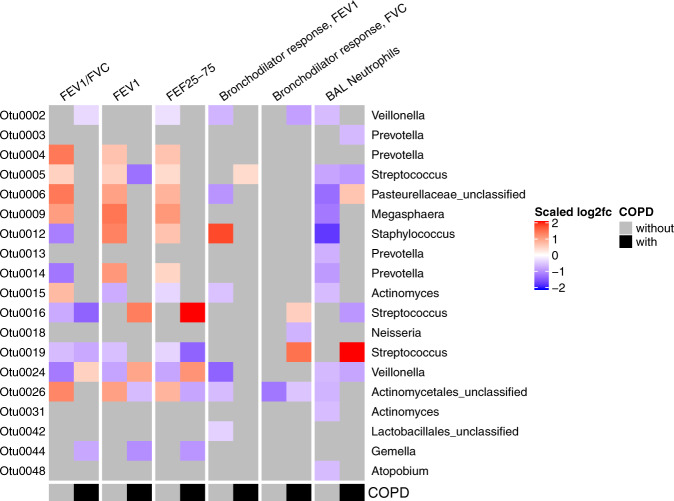
Heatmap summarizing results of DESeq analysis showing lung bacterial taxa significantly associated (*q*-value < 0.10) with measures of lung function, symptoms (CAT score), and percentage of BAL neutrophils. Further information is provided in Supplementary Table 3.

To complement these analyses, we examined if individual bacterial taxa were differentially abundant between the two groups of ever-smokers and/or associated with lung function measures within each group. Three specific taxa were differentially abundant by DESeq analysis between the ever-smokers without COPD and those with mild-moderate COPD: *Streptococcus* (OTU0005; log2FC = 2.05, *q* < 0.001), an unclassified *Lactobacillales* (OTU0042; log2FC = 1.47, *q* = 0.035), and *Veillonella* (OTU0024; log2FC = −1.41, *q* = 0.079; Fig. 5 and Supplementary Table 3). Among ever-smokers without COPD, the abundances of *Staphylococcus* (OTU0012), *Prevotella* (OTU0014), *Streptococcus* (OTU0016, OTU0019), and *Veillonella* (OTU0024) were negatively associated with lung function by FEV_1_/FVC ratio, FEV_1_ % predicted and/or FEF_25–75_. Of these taxa, *Streptococcus* OTU0016 and OTU0019 also negatively associated with lung function in the mild-moderate COPD group (FEV_1_/FVC ratio), along with *Streptococcus* OTU0005 (FEV_1_) and *Gemella* (OTU0044; FEV_1_/FVC ratio, FEV_1_ and FEF_25–75_). Taxa associated with greater bronchodilator response in either group included three *Streptococcus* taxa (OTU0005, OTU0016, and OTU0019) and a *Staphylococcus* (OTU0012). We also explored differential abundance relationships with leukocyte populations in the BAL (Supplementary Table 4), which were determined by flow cytometry as previously described^15^. Significant relationships were observed predominantly with the percentages of BAL neutrophils, wherein only *Streptococcus* OTU0019 and an unclassified member of the *Pasteurellaceae* family (OTU0006) were positively associated with BAL % neutrophils in the mild-moderate COPD group.

### Lung bacterial burden and cultivation experiments

Finally, we examined relationships between lung bacterial burden (log-16S rRNA copy numbers as proxy) and lung bacterial composition in BAL samples from all subjects in this study. Bacterial burden positively correlated with phylogenetic diversity of samples (Faith_PD; *r*_s_ 0.30, *p* < 0.001). Microbiota contributing to this relationship included members of *Veillonella* (*r*_s_ = 0.48), *Prevotella* (*r*_s_ = 0.28-0.37) and *Streptococcus* (*r*_s_ = 0.37) (all *p*_adj_ < 0.001). In contrast, the relative abundances of two *Pseudomonas* members and a *Staphylococcus* negatively correlated with bacterial burden (*r*_s_ = −0.21 to −0.26, *p*_adj_ < 0.001). We noted slightly higher bacterial burden in current smokers compared to subjects not currently smoking (median log-16S copies 4.6 ± 0.6 vs. 4.4 ± 0.7; Wilcoxon rank-sum *p* = 0.045). Bacterial burden and within-sample diversity measures did not differ between the groups.

Because of biological interest in several of the identified bacteria in this study (e.g. *Pseudomonas, Streptococcus and Staphylococcus* taxa), further efforts to identify the particular species were pursued. For *Pseudomonas* (OTU0007) which was associated with community variation along PC1, the representative sequence demonstrated little similarity to the *P. aeruginosa* represented in the mock community DNA. BLAST analysis of the representative sequence for OTU0007 also did not match *P. aeruginosa*, but rather a range of other species including *P. fluorescens* complex.

We also attempted to isolate by culture and identify the most prevalent *Staphylococcus* and *Streptococcus* species in our dataset (e.g. OTUs 0005, 0012, 0019). From unprocessed frozen (−80 °C) bronchial wash fluid, single colony isolates of Gram-positive cocci were sub-cultured and sequenced using primers for the full-length 16S rRNA gene, plus the *rnpB* locus (RNA subunit of the bacterial endoribonuclease P complex), as the latter can better differentiate *Streptococcus* species^16^. BLAST analysis of these sequences indicated these cultured isolates to be *Staphylococcus hominis and Streptococcus salivarius* (>97% similarity), which by CLUSTAL alignment were identified as OTU0012 and OTU0019, respectively.

## Discussion

In this sub-cohort from SPIROMICS, enriched in ever-smokers without a clinical diagnosis of COPD or with mild-moderate COPD, we observed previously unrecognized relationships between the compositional structure of lung microbiota, the relative abundances of specific bacterial members, and pathophysiologic attributes of COPD, in particular measures reflective of airways dysfunction. Variation in overall lung bacterial community structure did not associate with a categorical diagnosis of COPD, and only two bacterial taxa were significantly positively associated with COPD status (*Streptococcus* and *Lactobacillales*). Instead, we observed more associations between lung bacterial composition and measures of lung function, which provide more granular readouts of airway physiology. Given the milder disease in this cohort, the relationships between these indicators of airways dysfunction and lung bacterial composition hint at interactions in the lung environment that may contribute to airway disease in COPD. To identify specific bacteria that may be involved, we performed taxon-level differential abundance analyses. Although this entailed many comparisons with the lung function measures captured in SPIROMICS, results adjusted for false discovery identified several bacteria negatively associated with FEV_1_/FVC, FEV_1_ or FEF_25–75_ that have previously been implicated in chronic inflammatory airway diseases (e.g. *Streptococcus, Staphylococcus, Prevotella*, *Gemella*)^17,18^. We also noted relationships between lung bacterial composition and patient-centered measures of respiratory symptom burden (CAT score), suggesting possible clinical consequences related to lung dysbiosis in earlier stages of COPD. Together these results, from one of the largest bronchoscopy-based multicenter investigations focused on understanding the pathogenesis of COPD^14^, suggest that the lung microbiota present in those at risk for COPD may play a role in the pathogenesis of airways dysfunction.

An important strength of our study is the bronchoscopy-based and multicenter nature of our investigation, performed at clinical sites representing the U.S. geographic spectrum. Our study also contrasts from many other studies of the airway microbiome in COPD, where more often sputum has been analyzed to infer insights into the lung microbiome and in patients with more severe COPD or frequent exacerbation history^19–22^. These clinical features were neither the focus of this study nor characteristic of this cohort. We also analyzed cellular BAL which has been shown to capture greater representation of the lung bacterial community and differ in lung bacterial composition compared to acellular samples^23^. It is noteworthy that our data did not reveal differences in lung bacterial composition attributable to geographic locale, season in which bronchoscopy was performed, or current smoking status. An earlier multicenter study of the lung microbiome also did not find an association between current smoking and lung bacterial community structure^24^. We did observe slightly higher lung bacterial burden among current smokers, and it has been reported that BAL from smokers may promote the growth of specific bacterial species^25^. Our study was unique also in our attempt to culture primary isolates of organisms of interest identified in our analyses (e.g. *Staphylococcus, Streptococcus*). This led to the identification of two species whose sequences aligned to specific OTUs that may play a role in airways dysfunction. Although *Staphylococcus* and *Streptococcus* are known inhabitants of the upper airways, that these species were viably recovered from BAL samples and not identified as contaminants in our analysis, suggests they have the potential to interact actively in the lung environment.

Our study has a number of limitations. First, it is a descriptive analysis of the lung microbiota in COPD. Our focus on earlier stage disease, however, renders our study somewhat unique. Second, we did not confirm previously reported relationships between inhaled corticosteroid use and lung bacterial community structure in COPD^2^. This may be due to very few subjects identified as taking an inhaled steroid around the time of bronchoscopy, potentially limiting this evaluation. Similarly, robust examination of lung microbiota relationships to prospective exacerbation events in SPIROMICS subjects, while of interest, was not possible given the limited number of events in the timeframe of available data. Third, due to logistical challenges we could not directly characterize functional features of the microbiota, nor did we examine the identity of non-bacterial members of the lung microbiome. These topics are of interest to advance understanding of interactions between microbial kingdoms and with host immunity. We had limited paired immunological data available for this analysis but were able to explore relationships to BAL leukocyte numbers, which revealed a significant relationship between *Streptococcus* and BAL neutrophils. Future studies incorporating such data from the same biological compartment could be insightful, potentially supplementing the modest strength of the associations we observed with individual bacterial taxa. Indeed, efforts are underway in SPIROMICS to examine these aspects including possible functional interactions between the lung microbiota and host. Lastly, the current study was solely a cross-sectional analysis. While logistically challenging, longitudinal evaluations underway in the current phase of SPIROMICS will permit deeper evaluation of lung microbiota-immune interactions over time and how they relate to COPD development or progression.

The relationships we observed between the lung microbiota and measures of lung function, in particular BDR, FEF_25–75_ and PEFR, will also require further study. As with analogous findings in idiopathic pulmonary fibrosis^26,27^, a different lung disease linked in part to smoking, longitudinal studies of COPD could provide stronger evidence of the directionality of these associations and whether particular implicated microbiota precede rather than result from loss of lung function. Molecular mechanisms for the microbial associations with BDR are at present unclear, but this consistent finding in our analyses regardless of the inclusion of never-smokers argues for potential relevance to COPD pathophysiology. BDR measures can be variable over time, but in our subjects were not significantly different between their baseline visit and closest annual study visit before bronchoscopy. We observed similar associations with BDR when we used the baseline measures (not shown). Prior studies have reported relationships between airway dysbiosis and the presence of wheeze or airway hyper-reactivity in asthmatic subjects^28,29^, including involvement of *Streptococcus* and *Staphylococcus* species^29^. A recent analysis from COPDGene^30^, a more severe disease cohort, found that the presence of BDR was associated with radiographic evidence of small airway disease and more exacerbations.

Overall, the relationships observed lead us to speculate that airflow obstruction results in or could also reflect a mutually reinforcing relationship within microenvironmental conditions that exert selective pressure on the compositional make-up and functional behavior of microbiota in the niche. We previously reported that total mucin concentrations in sputum inversely associated with FEF_25–75_ in SPIROMICS subjects^31^, which may reflect one aspect of changes in the lung environment that could shape the microbiome. COPD is also associated with loss of several host defense factors that may facilitate bacterial invasion of airway epithelium^32–34^. Over time, evolving interactions between host and microbiota may provoke further immune responses and contribute to perpetuation of airway inflammation. Synergistic interactions between microbiota, as has been shown for *Streptococcus* and *Veillonella* species^35–38^, may also contribute. This ecological perspective of host-microbial interactions aligns with the “vicious cycle hypothesis” of inflammation and ‘infection’ in COPD^39^ and is important to maintain in considering how such interactions may affect airway disease pathogenesis in COPD. Findings from this study suggest additional avenues for further translational and mechanistic investigations into lung microbiota-host interactions in early stage COPD.

## Methods

### Subject characterization and research bronchoscopy

SPIROMICS is a multicenter observational study (NCT01969344; clinicaltrials.gov) of never-smokers, smokers without airflow obstruction or with mild, moderate or severe COPD. All SPIROMICS participants (*n* = 2981) underwent detailed clinical characterization and collection of biological and radiographic data, as previously described^6,40^. The institutional review boards of all SPIROMICS sites approved the main study protocol, and all participants provided written informed consent. Pulmonary function testing was performed according to the 2005 ATS/ERS guidelines. Ever-smokers were defined as current or former smokers with a smoking history of ≥20 pack-years; former smokers were those who had been free from use of any tobacco products for 6 months preceding the bronchoscopy. A subset of subjects (*n* = 215; inclusive of never-smokers, smokers with normal lung function, mild-moderate COPD, and severe COPD) additionally participated in a bronchoscopy sub-study^14^, which was approved by the institutional review boards of the participating SPIROMICS sites and for which subjects provided separate informed consent. The institutions that enrolled subjects for the research bronchoscopy included Columbia University/Weill Cornell Medical College, National Jewish Health (Denver, Colorado), University of Alabama at Birmingham, University of California at Los Angeles, University of California at San Francisco, University of Michigan, University of Utah, and Wake Forest University School of Medicine. Bronchoscopy was performed a median of 62 days after the preceding annual study visit (Supplementary Fig. 1), from which most of the clinical measures used in our analyses were obtained and did not differ significantly from earlier measurements (Supplementary Table 1). Samples of BAL allocated for microbiome analysis (10 mL into RNALater) were obtained from 181 subjects, along with biological control specimens (e.g. oral wash/tongue scraping, instrument samples such as bronchoscope suction channel flushes with sterile saline, negative controls of sterile saline or lidocaine). Remaining BAL was saved for other purposes in SPIROMICS including a 5-mL aliquot for potential microbial cultivation experiments. Samples were stored long-term at −80 °C until processing for this study.

### Sample processing, microbiota sequencing, and raw data processing

Five mL of BAL, oral rinse/tongue scraping, and bronchoscope flushes were centrifuged at high-speed (13,000 rpm) to pellet cellular material. Total genomic DNA was extracted from cell pellets using a commercial kit (DNeasy Blood and Tissue kit, Qiagen) with the following modifications: after re-suspension in ATL buffer the samples were transferred to Lysing Matrix E tubes (MP Biomedicals) and subjected to bead beating for 30 s before proceeding. The manufacturer’s protocol was modified to use 40 μL proteinase K instead of the recommended 20 μL, and samples were eluted with 100 μL of buffer AE instead of the suggested 200 μL. Negative extraction controls were generated by performing the extraction protocol without addition of sample but with sterile PBS.

The Microbial Systems Molecular Biology Laboratory (Microbiome) Core at the University of Michigan performed 16S rRNA gene (V4 region) sequencing using barcoded dual-index primers^41^ on an Illumina MiSeq (Illumina, San Diego, CA). Given the low biomass of samples, we employed a touchdown PCR amplification strategy^42^, including a mock reference community (ZymoBIOMICS; Zymo Research; Irvine, CA). Library preparation/sequencing controls (including elution buffer, sterile water, PBS, empty wells) also were sequenced. Several of these were performed in replicate. A mock community (Zymo) was included for quality control. Normalized pooled libraries were sequenced on the Illumina MiSeq platform using the 500 cycle MiSeq V2 Reagent kit. For internal testing purposes, BAL lavage samples were sequenced in triplicate (batch 2 only), while all other samples were sequenced once. The distribution of subjects by SPIROMICS group assignment did not differ between the two runs (Fisher exact test *p* > 0.05).

16S rRNA gene sequence reads were processed using mothur (v1.40.5)^43^, aligned to the SILVA reference alignment (release 102)^44^ and classified using the RDP 16S rRNA reference training set (version 16)^45^. The range of read counts in samples was 577 to 53,155, with the average being 21,444. Operational taxonomic units (OTUs) were defined using 97% similarity threshold.

Results of negative control samples (raw data prior to decontamination analysis using R package *decontam*^46^) are provided as Supplementary Table 5 and Supplementary Fig. 2. The prevalence method in *decontam* was used. The two batches of sample runs were decontaminated separately based on the negative controls collected/generated in parallel for the samples in a given batch. Initially, there were a total of 5172 OTUs and 986 OTUs were removed after *decontam* analysis. Eight of these OTUs were in common between the two decontamination runs (Otu0056, Otu0300, Otu0078, Otu1812, Otu0111, Otu0154, Otu0047, and Otu0204) and OTU 0011 was also removed because it appeared to be a contaminant. This brought the total of OTUs to 4193 prior to filtering by relative abundance. To compare further the two batches of samples, it was necessary to choose a single replicate sample from each subject in batch 2. For each patient, the geometric mean of normalized OTU counts was calculated to create a representative community to compare samples against. Then the Bray–Curtis distance between each individual sample and the mean sample was calculated and the sample with the lowest distance to the representative sample kept.

The count table, taxonomy table and phylogenetic tree file (*picante*)^47^ were imported into R for analysis using *phyloseq* (v1.26.0)^48^. For alpha-diversity calculations, reads were subsampled to the lowest count (577). All other analyses were performed with non-subsampled data, using relative abundance. The OTUs were filtered by average relative abundance >0.1% (final total of 125 OTUs analyzed). Principal component analysis of Hellinger-transformed abundance data from 84 paired BAL and oral rinse/tongue scrape samples demonstrated distinct segregation (clustering) of the two sample types (Supplementary Fig. 3; PERMANOVA *p* < 0.05).

### Statistical/data analysis

Clinical data from the most recent annual SPIROMICS visit preceding bronchoscopy were used wherever possible (SPIROMICS Core dataset CORE5_20180910). For exploratory analyses, we also included information available only from the baseline SPIROMICS visit. Non-parametric tests and Fisher’s exact tests were used where appropriate. Spearman correlation (*r*_s_) was used to compare continuous measures including alpha-diversity indices, with Benjamini–Hochberg correction for multiple comparisons^49^. To identify variables associated with bacterial community differences between subjects (i.e. beta-diversity), univariate distance-based PERMANOVA^50,51^ based on Bray–Curtis or weighted Unifrac distances was performed (*vegan* v2.5-4^27^), with 1000 permutations of the input matrices for determination of significance. The Unifrac distance incorporates phylogenetic information to capture the relatedness of bacterial communities between samples. Taxon-level differential abundance was determined by DESeq2 which utilizes a negative binomial generalized linear model with Wald’s test^52^.

### Assessment of bacterial burden and culture-based studies

Copy numbers of the 16S rRNA gene were assessed by quantitative (real-time) PCR using a standard curve comprising a 10-fold dilution series from 10^8^ to 10^2^ copies per microliter. TaqMan reagents (ABI) were used to amplify 3 µL of DNA; nuclease-free water was used in place of DNA for a no-template control. All reactions were performed in triplicate. Primers and probe were as follows: forward primer (TGG AGC ATG TGG TTT AAT TCG A), reverse primer (TGC GGG ACT TAA CCC AAC), probe (FAM-CAC GAG CTG ACG ACA CCC ATG CA-BHQ). Amplification was performed on the ABI Step One Plus thermocycler and data analyzed using Step One software version 2.1. To further identify primary species of interest (e.g. *Streptococcus*, *Staphylococcus*), aliquots of raw frozen BAL were cultured onto blood agar and in aerobic broth (sBHI medium) at 37 °C overnight. followed by sub-culture of individual colonies which by Gram stain revealed Gram-positive cocci in chains and clusters. Fresh liquid cultures were grown from single colonies and DNA isolated. Sequencing of the 16S ribosomal RNA gene and alignment in BLAST indicated two isolates in the genus *Streptococcus* and one isolate of *Staphylococcus hominis*. CLUSTAL (https://www.ebi.ac.uk/Tools/msa/clustalo/) was used to align the latter sequence with the representative sequences obtained from the BAL community and identify *Staphylococcus hominis* (OTU0012). Due to high sequence similarity in the 16S rRNA gene for the genus *Streptococcus*, sequencing of the *rnpB* locus was performed^16^ to determine species identity.

### Reporting summary

Further information on research design is available in the Nature Research Reporting Summary linked to this article.

## Supplementary information

Supplementary Information

Reporting Summary

## Data Availability

All data supporting the findings of this study are available within the paper and in the Supplementary Information. The 16S rRNA gene sequence data have been submitted to the NCBI Sequence Read Archive (SRA) under BioProject ID PRJNA673153. These data also are available from the SPIROMICS Genomics and Informatics Coordinating Center (https://www2.cscc.unc.edu/spiromics/contact-gic).

## Code availability

Information regarding software, data inputs and code used to support the findings of this study are in the Reporting Summary. No custom code was developed for this study. The specific code implemented, based on published analysis packages, can be obtained from the authors upon request.
