# Supplementary material for: Lung microbiota associations with clinical features of COPD in the SPIROMICS cohort
Source: NPJ Biofilms Microbiomes. 2021 Feb 5;7:14. doi: 10.1038/s41522-021-00185-9 (PMC7865064; doi:10.1038/s41522-021-00185-9)
Supplement: Supplementary file 2 — Reporting Summary [file 41522_2021_185_MOESM2_ESM.pdf]

nature research

Corresponding author(s): Huang, Yvonne

Last updated by author(s): Jan 6, 2021

Reporting Summary

Nature Research wishes to improve the reproducibility of the work that we publish. This form provides structure for consistency and transparency in reporting. For further information on Nature Research policies, see our Editorial Policies and the Editorial Policy Checklist.

Statistics

For all statistical analyses, confirm that the following items are present in the figure legend, table legend, main text, or Methods section.

n/a	Confirmed	
☐	☒	The exact sample size (*n*) for each experimental group/condition, given as a discrete number and unit of measurement
☐	☒	A statement on whether measurements were taken from distinct samples or whether the same sample was measured repeatedly
☐	☒	The statistical test(s) used AND whether they are one- or two-sided<br>*Only common tests should be described solely by name; describe more complex techniques in the Methods section.*
☐	☒	A description of all covariates tested
☐	☒	A description of any assumptions or corrections, such as tests of normality and adjustment for multiple comparisons
☐	☒	A full description of the statistical parameters including central tendency (e.g. means) or other basic estimates (e.g. regression coefficient) AND variation (e.g. standard deviation) or associated estimates of uncertainty (e.g. confidence intervals)
☐	☒	For null hypothesis testing, the test statistic (e.g. *F*, *t*, *r*) with confidence intervals, effect sizes, degrees of freedom and *P* value noted<br>*Give P values as exact values whenever suitable.*
☒	☐	For Bayesian analysis, information on the choice of priors and Markov chain Monte Carlo settings
☒	☐	For hierarchical and complex designs, identification of the appropriate level for tests and full reporting of outcomes
☒	☐	Estimates of effect sizes (e.g. Cohen's *d*, Pearson's *r*), indicating how they were calculated

Our web collection on statistics for biologists contains articles on many of the points above.

Software and code

Policy information about availability of computer code

Data collection	The 16S rRNA gene sequence data were generated on an Illumina MiSeq using MiSeq reagent kit v2 and MiSeq Control Software version 2.6.2.1
Data analysis	Analysis steps are as detailed in the supplemental material. No custom code was used. Specific packages/versions used included:<br>mothur 1.40.5<br>R version 3.5.1 (2018-07-02) -- "Feather Spray"<br>decontam 1.8.0 (with https://benjjneb.github.io/decontam/vignettes/decontam_intro.html)<br>phyloseq 1.26.0 (with https://github.com/DenefLab/MicrobeMiseq/blob/master/R/miseqR.R)<br>mvabund 4.0.1<br>vegan 2.5.6

For manuscripts utilizing custom algorithms or software that are central to the research but not yet described in published literature, software must be made available to editors and reviewers. We strongly encourage code deposition in a community repository (e.g. GitHub). See the Nature Research guidelines for submitting code & software for further information.

Data

Policy information about availability of data

All manuscripts must include a data availability statement. This statement should provide the following information, where applicable:

- Accession codes, unique identifiers, or web links for publicly available datasets
- A list of figures that have associated raw data
- A description of any restrictions on data availability

The data supporting the findings of this study are included in the published paper and the Supplementary Information and files. The 16S rRNA gene sequence data

have been submitted to the NCBI Sequence Read Archive (SRA) under BioProject ID PRJNA673153

Field-specific reporting

Please select the one below that is the best fit for your research. If you are not sure, read the appropriate sections before making your selection.

☒ Life sciences ☐ Behavioural & social sciences ☐ Ecological, evolutionary & environmental sciences

For a reference copy of the document with all sections, see nature.com/documents/nr-reporting-summary-flat.pdf

Life sciences study design

All studies must disclose on these points even when the disclosure is negative.

Sample size	The published study is a cross-sectional analysis of ever-smokers without or with mild-moderate COPD, who were enrolled in a prospective observational cohort study (SPIROMICS parent trial NCT01969344). Sample size was therefore based on matched lung sample and data availability.
Data exclusions	No relevant exclusions.
Replication	Only one BAL sample for designated microbiome analysis was available from each participant in this single cohort study.
Randomization	The parent study from which the samples and clinical data were collected was a non-randomized prospective observational cohort.
Blinding	The parent SPIROMICS bronchoscopy sub-study employed an a priori enrollment strategy to ensure adequacy of group representation to address main research questions of interest. Subjects were recruited accordingly based on target numbers for each group as detailed in Wells et al. [Safety and Tolerability of Comprehensive Research Bronchoscopy in Chronic Obstructive Pulmonary Disease. Results from the SPIROMICS Bronchoscopy Substudy. Ann Am Thorac Soc 16, 439–446 (2019)]. Technical staff at the University of Michigan were blinded to group assignment during sample processing for microbiota data generation. After initial raw data processing to generate the final OTU table, subsequent analyses were performed without blinding, since the study goal was to determine associations with COPD status and COPD-related clinical features.

Reporting for specific materials, systems and methods

We require information from authors about some types of materials, experimental systems and methods used in many studies. Here, indicate whether each material, system or method listed is relevant to your study. If you are not sure if a list item applies to your research, read the appropriate section before selecting a response.

Materials & experimental systems

n/a	Involved in the study	
☒	☐	Antibodies
☒	☐	Eukaryotic cell lines
☒	☐	Palaeontology and archaeology
☒	☐	Animals and other organisms
☐	☒	Human research participants
☐	☒	Clinical data
☒	☐	Dual use research of concern

Methods

n/a	Involved in the study	
☒	☐	ChIP-seq
☒	☐	Flow cytometry
☒	☐	MRI-based neuroimaging

Human research participants

Policy information about studies involving human research participants

Population characteristics	As presented in Table 1 of the study, a total of 181 adult subjects were included. This included 24 never-smokers, 79 ever-smokers without COPD (normal FEV1/FVC ratio), 71 ever-smokers with mild/moderate COPD, and 7 with severe COPD, groups as defined in the parent SPIROMICS study and bronchoscopy sub-study. 52% of the 181 subjects were male. COPD subjects were slightly older.
Recruitment	As described in Wells et al. [Ann Am Thorac Soc 16, 439–446 (2019], the SPIROMICS bronchoscopy study employed an a priori enrollment strategy to ensure adequacy of control groups to address main research questions of interest. Accordingly, target numbers for each group were established. BAL samples analyzed in this study represented 89%, 87%, 80% and 88% of the subjects enrolled into each group in the bronchoscopy sub-study.
Ethics oversight	Each SPIROMICS clinical site participating in the bronchoscopy sub-study received institutional IRB approval for the research bronchoscopy study. The same protocol was used at all sites. The SPIROMICS Genomics and Informatics Coordinating Center

provides oversight for all aspects of SPIROMICS investigations. All bronchoscopy participants provided voluntary informed written consent to undergo research bronchoscopy.

Note that full information on the approval of the study protocol must also be provided in the manuscript.

Clinical data

Policy information about clinical studies

All manuscripts should comply with the ICMJE guidelines for publication of clinical research and a completed CONSORT checklist must be included with all submissions.

Clinical trial registration	SPIROMICS parent study; NCT01969344
Study protocol	Protocol information has been published/provided under the following publications (Pubmed-PMCID): PMC6441692 and PMC3954445. Further information if needed can be obtained from the SPIROMICS GIC (https://www2.cscc.unc.edu/spiromics/)
Data collection	All clinical data was collected at the participating SPIROMICS sites, as described in the above publications. Data was sent to the SPIROMICS Genomics and Informatics Coordinating Center for storage, curation and provision to investigators.
Outcomes	The selected clinical variables that were the focus of the lung microbiota association analyses were those of clinical relevance or that reflect COPD pathophysiology and morbidity. Thus, COPD status/severity as well as validated measures of lung function and symptoms were examined.
